# Quantifying the Variation in the Geometries of the Outer Rims of Corolla Tubes of *Vinca major* L.

**DOI:** 10.3390/plants11151987

**Published:** 2022-07-30

**Authors:** Lin Wang, Qinyue Miao, Ülo Niinemets, Johan Gielis, Peijian Shi

**Affiliations:** 1 College of Science & College of Biology and the Environment, Nanjing Forestry University, Nanjing 210037, China; lwang@njfu.edu.cn (L.W.); qymiao@njfu.edu.cn (Q.M.); 2 Institute of Agricultural and Environmental Sciences, Estonian University of Life Sciences, 51006 Tartu, Estonia; 3 Estonian Academy of Sciences, 10130 Tallinn, Estonia; 4 Department of Biosciences Engineering, University of Antwerp, B-2020 Antwerp, Belgium; johan.gielis@uantwerpen.be

**Keywords:** flower geometry, Gielis equation, model complexity, natural geometries, planar coordinates, polygonal structure, radial symmetry

## Abstract

Many geometries of plant organs can be described by the Gielis equation, a polar coordinate equation extended from the superellipse equation, $r=a{\left[|\cos\left(\frac{m}{4}\varphi \right){|}^{n_{2}}+|\frac{1}{k}\sin\left(\frac{m}{4}\varphi \right){|}^{n_{3}}\right]}^{-1/n_{1}}$. Here, *r* is the polar radius corresponding to the polar angle φ; *m* is a positive integer that determines the number of angles of the Gielis curve when φ ∈ [0 to 2π); and the rest of the symbols are parameters to be estimated. The pentagonal radial symmetry of calyxes and corolla tubes in top view is a common feature in the flowers of many eudicots. However, prior studies have not tested whether the Gielis equation can depict the shapes of corolla tubes. We sampled randomly 366 flowers of *Vinca major* L., among which 360 had five petals and pentagonal corolla tubes, and six had four petals and quadrangular corolla tubes. We extracted the planar coordinates of the outer rims of corolla tubes (in top view) (ORCTs), and then fitted the data with two simplified versions of the Gielis equation with *k* = 1 and *m* = 5: $r=a{\left[{\left|\cos\left(\frac{5}{4}\varphi \right)\right|}^{n_{2}}+{\left|\sin\left(\frac{5}{4}\varphi \right)\right|}^{n_{3}}\right]}^{-1/n_{1}}$ (Model 1), and $r=a{\left[{\left|\cos\left(\frac{5}{4}\varphi \right)\right|}^{n_{2}}+{\left|\sin\left(\frac{5}{4}\varphi \right)\right|}^{n_{2}}\right]}^{-1/n_{1}}$ (Model 2). The adjusted root mean square error (RMSE_adj_) was used to evaluate the goodness of fit of each model. In addition, to test whether ORCTs are radially symmetrical, we correlated the estimates of *n*_2_ and *n*_3_ in Model 1 on a log-log scale. The results validated the two simplified Gielis equations. The RMSE_adj_ values for all corolla tubes were smaller than 0.05 for both models. The numerical values of *n*_2_ and *n*_3_ were demonstrated to be statistically equal based on the regression analysis, which suggested that the ORCTs of *V. major* are radially symmetrical. It suggests that Model 1 can be replaced by the simpler Model 2 for fitting the ORCT in this species. This work indicates that the pentagonal or quadrangular corolla tubes (in top view) can both be modeled by the Gielis equation and demonstrates that the pentagonal or quadrangular corolla tubes of plants tend to form radial symmetrical geometries during their development and growth.

## 1. Introduction

In geometry, the circle and ellipse have been demonstrated to be expressed as two special cases of the superellipse [1]. As a direct extension of the superellipse equation in the polar coordinate system, Gielis [2,3] created a highly versatile equation to reflect natural geometries, especially symmetrical geometries. We refer to it as the Gielis equation hereinafter. In the past decade, many studies have been carried out to examine the validity of the Gielis equation in fitting actual biological geometries. A simplified version of this equation was used by Shi et al. [4,5] and Lin et al. [6] to fit the boundary data of cross-sections of tree rings for five species of conifers and bamboo leaves for 46 bamboo species. These studies verified the potential of the Gielis equation to describe the shapes of tree rings and bamboo leaves and demonstrated that the Gielis equation is a valid scientific method for quantitative characterization of the size and shape of widely differing planar biological objects.

Shi et al. [7] proposed a twin version of the Gielis equation by introducing a link function and found that the twin Gielis equation was superior in depicting the shapes of some sea stars. Tian et al. [8] used the Gielis equation to fit the seed projections (in side view) of two *Gingko biloba* cultivars, and used it to quantify the morphological differences between the two cultivars. Li et al. [9] compared the original Gielis equation with its twin version in describing the planar projections of *Koelreuteria paniculata* fruits (in top view) and demonstrated that the two versions of the Gielis equation both can model the shapes of the vertical fruit projections well. Nevertheless, the twin Gielis equation predicted an axial symmetry of *K. paniculata* fruits, while the original Gielis equation tends to overfit the data. In addition, a recent study shows that a simplified Gielis equation can describe all existing egg shapes of birds and has a better goodness of fit than other egg shape models [10]. Flowers can also be modelled in the same way [2,3,11,12,13], but the capacity of the Gielis equation to simulate flower shape has not been quantitatively studied.

Here, we focused on the geometries of the outer rims of corolla tubes of *Vinca* flowers that are representative to the five-petal flowers with a fused pentagonal base (corolla top). The genus *Vinca* is native to western Mediterranean Europe, Asia Minor and Northern Africa, but it has been introduced as ornamental to all continents and has naturalized in many sites. It is a small evergreen ground cover plant and in addition to sexual reproduction, spreads via stolons. Taxonomically, *Vinca* belongs to the Apocynaceae family, one of the five families within the order Gentianales; together with the orders Solanales and Lamiales they form the Lamiids [14]. Lamiids are characterized by late sympetaly, the fusion of stamen filaments with the corolla tube and opposite leaves [15]. The Apocynaceae have a conserved architecture of highly synorganized flowers, and within this family *Vinca* L. is the type genus of the tribe Vinceae, in particular, of the subtribe Vincinae. The corolla is infundibuliform, and the lobe aestivation is sinistrorse [15].

The petals of *Vinca* are fused at their bottom, forming a corolla tube. When the flower opens, the distal, unfused parts of the petals fold back in a plane, whereas the upper part of the corolla tube formed by the fused parts of the petals becomes clearly delineated. The upper ridge or rim of the corolla tube has a clear pentagonal symmetry (although quadrangular or hexagonal symmetry may occur). In contrast to the purple color of the free petals and the base of the tube, the upper rim of the floral tube is white. The purple petals exhibit a high ultraviolet reflectance, whereas the corolla tube, in particular, the upper rim of the flower, strongly absorbs ultraviolet (Ultraviolet Flowers: Vinca minor. Available online: http://www.naturfotograf.com/UV_VINC_MIN.htm (accessed on 1 July 2022)).

**Figure 1:**
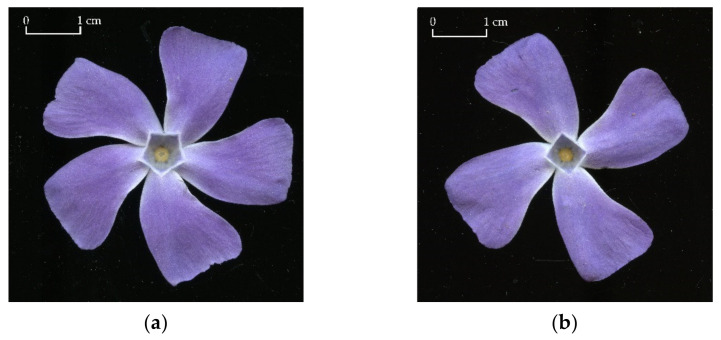
Representative flowers of *Vinca major* L. with five (**a**) and with four petals (**b**).

Most flowers in this species have five petals, but flowers with four or six petals occur rarely (Figure 1). Five petals correspond to a corolla tube of pentagonal symmetry and a pentagonal rim, and four petals correspond to a quadrangular rim (Figure 1). In the remainder of the paper, we use the term the outer rim of the corolla tube (in top view) (ORCT) to denote this polygonal structure and characterize the geometry of the outer rim of the corolla tube in top view (i.e., represented by a vertical projection). The two types of ORCTs (i.e., pentagonal and quadrangular rims) both seem to exhibit a radial symmetry, whereas the distal ends of the petals are rotated counterclockwise, relative to the corolla tube. The main reason is the sinistrorse aestivation of the flowers. The *Vinca* flowers display contorted aestivation of the corolla, and each petal is asymmetric. However, the entire corolla exhibits a rotational symmetry (Figure 1).

Pentagonal symmetry is a general condition in Eudicots [16], but a clear pentagonal shape, as in the corolla tube of *Vinca*, is uncommon. In trumpet, campanulate or salverform corolla tubes, the transition from fused proximal parts of the petals to the free parts is gradually curved. In *Vinca*, on the other hand, the plane formed by the free ends of the petals is almost perpendicular to the corolla tube. Although the ORCT is never completely flat, a projection using a photography or an image scanner can be obtained, making a 2D quantitative study possible. In this study, 360 flowers with five petals and six flowers with four petals from *V. major* were sampled to examine whether the ORCTs follow the Gielis equation, and to evaluate whether a deviation from pure rotational symmetry can be found.

## 2. Materials and Methods

### 2.1. Flower Sampling and Image Processing

*Vinca major* flowers were randomly sampled at the Nanjing Forestry University campus (118°48′35″ E, 32°04′67″ N), Nanjing, China from 7–23 April 2022 when the peak blooming occurred. To keep the flowers fresh, each sampled flower was placed in a 10 mL beaker with 1–2 mL water until its image was scanned. The flowers were scanned by an Epson photo scanner (V550, Epson, Batam, Indonesia) at a resolution of 2400 dpi, then their images were converted into black-white images after being cropped and saved as bmp (Bitmap) format using Adobe Photoshop CS2 (version 9.0; Adobe, San Jose, CA, USA; http://www.adobe.com/products/photoshop.html, accessed on 1 July 2022).

### 2.2. Data Acquisition

To extract the planar coordinates of the outer rim of the corolla tube (in top view) (ORCT), we used MATLAB (version ≥ 2009a; MathWorks, Natick, MA, USA) with a program developed by refs. [4,17,18]. We fitted the coordinate data of each ORCT using the ‘biogeom’ package (version 1.0.5) [19] based on R (version 4.2.0) [20].

### 2.3. Models

Gielis [2] proposed a polar coordinate equation to describe natural shapes:

(1) $$r(\varphi )={\left({\left|\frac{1}{A}\cos\left(\frac{m}{4}\varphi \right)\right|}^{n_{2}}+{\left|\frac{1}{B}\sin\left(\frac{m}{4}\varphi \right)\right|}^{n_{3}}\right)}^{-\frac{1}{n_{1}}}$$

where *r* and φ are the polar radius and polar angle, respectively; *A*, *B*, *n*_1_, *n*_2_ and *n*_3_ are parameters to be fitted; and *m* is a positive integer that determines the number of angles of the Gielis curve within [0, 2π). Given that the ORCT can be pentagonal or quadrangular, *m* was either five or four in this study.

Equation (1) can be rewritten as [7,8]:

(2) $$r(\varphi )=a{\left({\left|\cos\left(\frac{m}{4}\varphi \right)\right|}^{n_{2}}+{\left|\frac{1}{k}\sin\left(\frac{m}{4}\varphi \right)\right|}^{n_{3}}\right)}^{-\frac{1}{n_{1}}}$$

where $a=A^{n_{2}/n_{1}}$ and $k=B/A^{n_{2}/n_{3}}$. In this work, *k* was set to 1 because the ORCT of *V. major* exhibits radial symmetry. Most flowers have five petals, so *m* was set to 5, resulting in:

(3) $$r(\varphi )=a{\left({\left|\cos\frac{5}{4}\varphi \right|}^{n_{2}}+{\left|\sin\frac{5}{4}\varphi \right|}^{n_{3}}\right)}^{-\frac{1}{n_{1}}}$$

To test whether a radial symmetrical version of the Gielis equation is applicable to the ORCT, we set *n*_2_ = *n*_3_ in Equation (3) so that we have:

(4) $$r(\varphi )=a{\left({\left|\cos\frac{5}{4}\varphi \right|}^{n_{2}}+{\left|\sin\frac{5}{4}\varphi \right|}^{n_{2}}\right)}^{-\frac{1}{n_{1}}}$$

We refer to Equations (3) and (4) as Models 1 and 2 for convenience hereinafter.

### 2.4. Model Fitting and Data Analysis

We used the Nelder–Mead optimization method [21] to minimize the residual sum of square (RSS) between the observed and predicted polar radii, and obtained the estimated values of the parameters in Equations (3) and (4):

(5) $$\mathrm{RSS}=\sum_{i=1}^{N}{\left(r_{i}-{\hat{r}}_{i}\right)}^{2}$$

where *r_i_* represents the observed distance from the polar point to the *i*-th point on a scanned ORCT; ${\hat{r}}_{i}$ represents the predicted distance from the polar point to the *i*-th point on the predicted ORCT based on Equation (3) or Equation (4); and *N* represents the number of data points on a scanned ORCT.

Additionally, the root-mean-square error (RMSE) was calculated to reflect the goodness of fit:

(6) $$\mathrm{RMSE}=\sqrt{\mathrm{RSS}/N}$$

However, given the influence of the ORCT size (area) on absolute values of RMSE, we used the adjusted RMSE (RMSE_adj_) [7,9,22]. In that case, we can directly compare the differences in the model goodness of fits among different ORCT sizes.

(7) $${\mathrm{RMSE}}_{\mathrm{adj}}=\sqrt{\frac{\mathrm{RSS}/N}{S/\pi }}$$

where *S* represents the area of an ORCT. The RMSE_adj_ represents the ratio of the mean absolute deviation (between the observed and predicted radii from the polar point to the ORCT) to the radius of a hypothetical circle whose area equals to that of the ORCT projection, which can standardize the prediction error regardless of the ORCT size.

There are still four parameters in Model 1, and the question is whether a less complex model can produce an approximate goodness of fit with fewer parameters. On the one hand, the difference between *n*_2_ and *n*_3_ in Model 1 determines the extent of symmetry. If the difference is equal to zero (i.e., Model 2), it can produce a perfectly axial symmetrical curve for the ORCT; the larger the difference, the worse the extent of the rim’s symmetry. On the other hand, the ORCTs of interest appear visually to be axial symmetrical pentagons, so it is necessary to test whether Model 1 (with four model parameters) can be simplified to Model 2 (with three model parameters). Therefore, we performed a linear regression on the estimated values of *n*_3_ and *n*_2_ for all of the samples. To stabilize the variance of the estimated values of the two parameters and to normalize the data, the log-transformation was used [23,24]:

(8) y=α+β x

where *y* = ln $\hat{n}$_3_ and *x* = ln $\hat{n}$_2_, where the circumflex represents the estimated value. The parameters α and β were estimated using reduced major axis regression protocols [25,26]. The bootstrap percentile method [27,28] was used to calculate 95% confidence intervals (CIs) of the intercept and the slope of the regression line. If the CI of the intercept includes zero and the CI of the slope includes one, it can indicate that *n*_3_ is not significantly different from *n*_2_, which means that Model 2 is superior to Model 1 due to reduced complexity. It also suggests that ORCTs tend to be perfectly axially symmetrical. If *n*_3_ is not statistically significant from *n*_2_, it is unnecessary to compare the RMSE_adj_ values of the two models. It is apparent that an additional parameter can increase the goodness of fit, but it might result from the overfitting to the data with a certain measurement error. In other words, the flexibility in curve fitting of Model 1 may cause an incorrect parameter estimation due to the measurement errors. As a rule of thumb, a ≤ 0.05 RMSE_adj_ can reflect the validity of a model in curve fitting.

To compare the goodness of fit between Models 1 and 2, a paired sample *t*-test was used to compare the average values of the RMSE_adj_ values of Models 1 and 2 at 0.05 significance level. The log-transformed values of adjusted RMSEs were used to normalize the data of adjusted RMSEs.

In addition, we used nonlinear least-squares to fit the ORCTs by minimizing the sum of squares (RSS) between the observed and predicted polar radii. The distribution of the residuals in nonlinear least-squares is usually hypothesized to be normal, but the hypothesis in nonlinear least-squares seldom holds true. In that case, we calculated the mean and corresponding 95% CI of residuals to examine whether the mean equals 0 and the corresponding 95% CI includes 0. We also calculated the skewness (*S_k_*) of residuals to see whether the skewness seriously deviates from zero. For a normal distribution, the skewness equals zero. A positive skewness represents a right skewed distribution curve, and a negative skewness represents a right-skewed distribution curve [17]:

(9) $$S_{k}=E\left[{\left(\frac{z-\mu }{\sigma }\right)}^{3}\right]$$

where *z* represents the residual between the *i*-th observed and predicted radii; μ represents the mean of residuals; and σ represents the standard error of residuals. We used the Shapiro–Wilk test [29] to test the normality of residuals only as a reference.

## 3. Results

**Figure 2:**
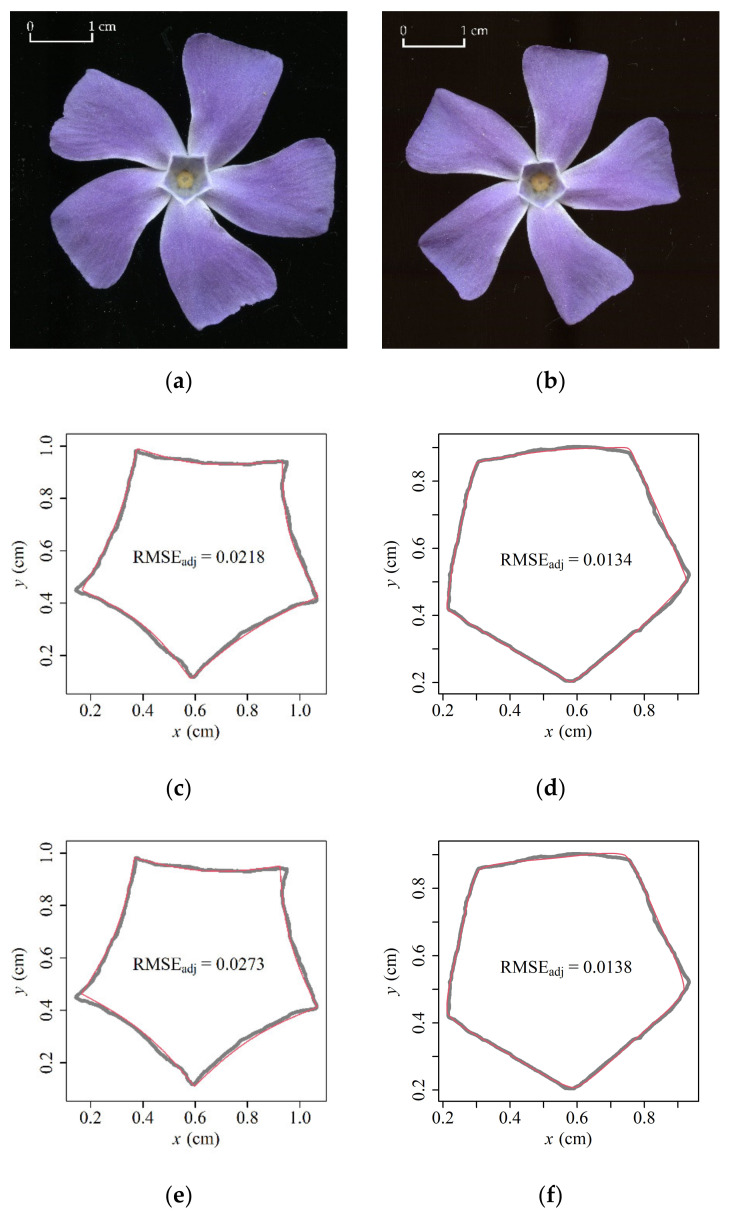
Representative images of *V. major* flowers with five petals (top view; **a**,**b**), and the measured (gray) and predicted (red) outer rims of corolla tubes (ORCTs) using Model 1 (**c**,**d**) and Model 2 (**e**,**f**). The boundary coordinate data of ORCTs were obtained from scanned images, and were fitted by the R package ‘biogeom’. RMSE_adj_ is the adjusted root-mean-square error (Equation (7)).

**Figure 3:**
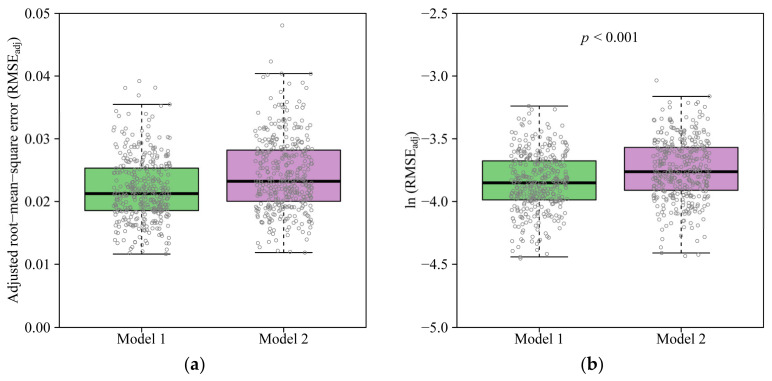
Comparison of adjusted root-mean-square errors (**a**, RMSE_adj_, Equation (7)) and their ln-transformations between Models 1 and 2 (**b**). The thick horizontal lines represent median values in the boxes; a box’s body length represents the difference between the 3/4 quantile and the 1/4 quantile; whiskers represent 1.5 times the box’s body length or maximum (or minimum) values; and the small gray open circles represent the distribution of data points. The ln-transformed values in (**b**) were compared by a paired sample *t*-test.

Both Models 1 and 2 provided generally a good representation of the ORCT (Figure 2 for sample fits). The RMSE_adj_ values of pentagonal ORCTs predicted by Models 1 and 2 ranged from 0.0116 to 0.0392 and from 0.0118 to 0.0481, respectively (Figure 3a). This verified the validities of the two models in describing the shapes of ORCTs of *V. major*. The RMSE_adj_ values calculated by Model 1 were smaller than those calculated by Model 2 for 346 out of 360 (ca. 96%) ORCT samples, and for the remaining 14 samples, the RMSE_adj_ values between the two models were the same. The mean ln(RMSE_adj_) of Model 1, *M*_1_, was significantly smaller than that of Model 2, *M*_1_ (*t* = −5.5666, df = 718, *p* < 0.001). This indicates that Model 1 with four parameters provided a better fit than Model 2 with three parameters, although the difference was significant in statistics but fairly small. The percentage error, i.e., $\left|\left(M_{2}-M_{1}\right)/M_{1}\right|\times 100\%$, between the two mean ln(RMSE_adj_) values, was less than 2.7% (Figure 3b). From the tradeoff between the model complexity and goodness of fit, Model 1 is not as good as Model 2, and the latter has a more concise model structure.

**Figure 4:**
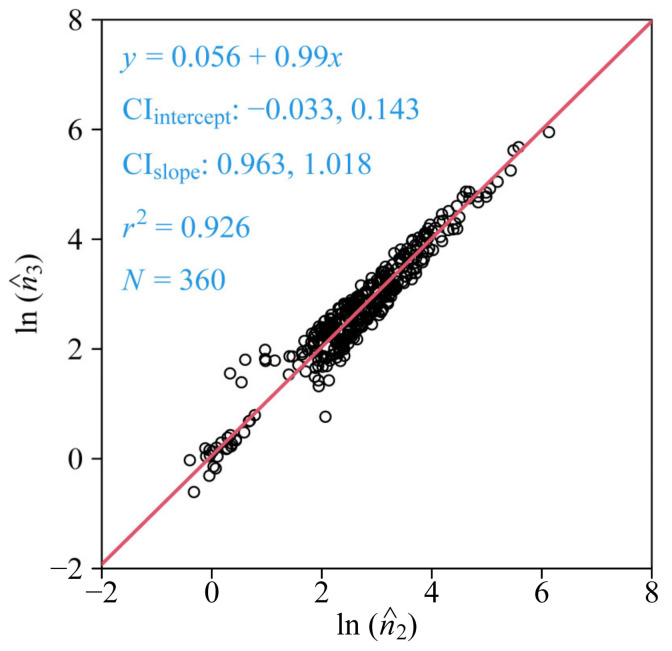
Fitted results to the data of the estimated values of the model parameter *n*_3_ vs. the model parameter *n*_2_ (Equation (3)). The data were fitted according to reduced major axis protocols on a log-log scale. *y* represents the ln-transformation of the estimated value of *n*_3_; *x* represents the ln-transformation of the estimated value of *n*_2_; the straight line is the regression line; CI_intercept_ represents the 95% confidence interval of the intercept; CI_slope_ represents the 95% confidence interval of the slope; *r*^2^ is the coefficient of determination that reflects the goodness of fit; and *N* is the sample size, i.e., the number of flowers used.

**Figure 5:**
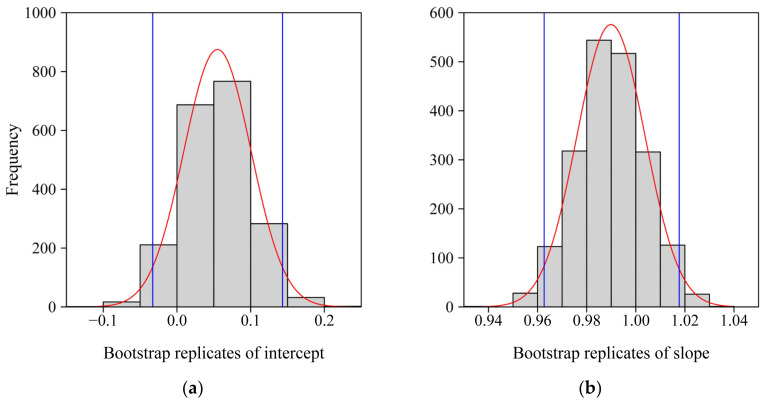
Frequency histograms of bootstrap replicates of the intercept (**a**) and slope (**b**) of the regression between the model parameters *n*_3_ and *n*_2_ (Equation (3); Figure 4). The red curves are normal density curves; and the blue vertical straight lines represent 0.025 quantile (left) and 0.975 quantile (right) that are the lower and upper bounds of the 95% confidence interval in each panel.

The results of the linear regression of the data of ln($\hat{n}$_3_) vs. ln($\hat{n}$_2_) and the distribution diagrams of 2000 bootstrap replicates of the regression intercept (α) and slope (β) indicated that the 95% CI of the intercept included zero and the 95% CI of the slope included 1.0 (Figure 4 and Figure 5). Thus, there was no significant difference between the estimated values of *n*_3_ and that of *n*_2_, suggesting that the three-parameter Model 2 is sufficient to depict the geometry of the ORCT of *V. major*, although the goodness of fit of Model 1 is slightly greater than that of Model 2 (Figure 3). The estimated values of parameters in Models 1 and 2 have been listed in Tables S1 and S2 in the online Supplementary Materials.

Although all *p* values of the normality test were smaller than 0.05, indicating that the distributions of residuals between the observed and predicted radii were not normal, the skewness only ranged from −1.1 to 1.1, which suggested that the distributions of residuals were not seriously skewed. The means of residuals for all samples ranged from −0.0002 to 0.0006 approximate to zero, and all of the 95% CIs of residuals included 0 (see Table S2). In other words, the distribution of residuals is approximate to be normal, and the above results at least demonstrated the validity of nonlinear least-squares in fitting the ORCTs.

## 4. Discussion

### 4.1. Analysis of the Prediction Errors

Multiple artificial measurement errors occurred when we scanned and extracted the boundaries of the 360 outer rims of corolla tubes (ORCTs). On the way from the site of plant growth to the laboratory, artificial physical pressures might have caused a certain deformation of the flowers. In addition, during scanning, the physical pressures on different contact points of a ORCT with the scanning plane could also cause a certain morphological deformation of the ORCT. Thus, there are some parts on an ORCT especially close to the vertices of the pentagon that are not well resolved in scanned images. This generates some uncertainties in extracting the ORCT. Nevertheless, given that such deformations were smaller than 0.05 mm, we conclude that the impact of such deformations on model fitting can be neglected.

In addition, the interspecific variation from a perfect symmetrical pattern in the growth process of *V. major* probably increased the deviation of the actual shape of ORCT from the predicted shape. The intraspecific variation reflects both genotypic differences as well as plastic modifications to differences in the growth microenvironment, including differences in the availability in light, water and nutrients [30,31,32,33,34]. Although the plants used were irrigated by sprinklers, the individuals closer to the water source formed a denser cover, indicating that water availability introduced some changes in the plant phenotype in this study. A stronger intraspecific competition for space at greater water availability caused some physical compression within adjacent individuals during flower growth; this resulted in the deformation of some flowers to a certain degree and subsequently led ORCTs to be less symmetrical. All of the factors outlined likely had some impact on the prediction errors using the two simplified Gielis models to describe the shapes of ORCTs. Nevertheless, the RMSE_adj_ values of the two models were all smaller than 0.05. This verified the validity of the Gielis equation in fitting the real natural geometries of corolla tubes of *V. major*.

### 4.2. Comparison of the Two Models

The parameters *n*_2_ and *n*_3_ determine the extent of symmetry for the geometry generated by Model 1. The regression analysis indicated that there was no significant difference between *n*_2_ and *n*_3_, indicating that the analysis can be simplified by replacing *n*_3_ with *n*_2_. Therefore, we conclude that Model 2 with fewer parameters can replace Model 1 for describing the ORCT of *V. major*. Although most of the adjusted RMSE values calculated by Model 1 were slightly smaller than those calculated by Model 2 among 360 ORCTs, all of the RMSE_adj_ values for both models were less than 0.05. This means that for any sample, the average absolute deviation between the observed and predicted radii from the polar point to an ORCT was less than 5% of the radius of a circle whose hypothetical area equals that of the ORCT.

The use of Model 2 has several practical advantages. In particular, as fewer parameters need to be fitted, the efficiency of parameter estimation is higher. This implies that the probability for the model parameters not to converge is lower for Model 2; also, the average computer running time to complete the optimization for each dataset using Model 2 is less than that using Model 1. In addition, Model 1 with an additional parameter *n*_3_ tends to lead to an overfitting of the planar coordinate data of ORCTs. Accordingly, this blurs the biological meaning of the parameter values. Model 2 with parameters *n*_3_ = *n*_2_ diminishes the possibility of generating an asymmetrical curve and therefore agrees better with previous studies (e.g., refs. [7,9]).

### 4.3. Variation in the Number of Polygon Sides of the ORCT of V. major

**Figure 6:**
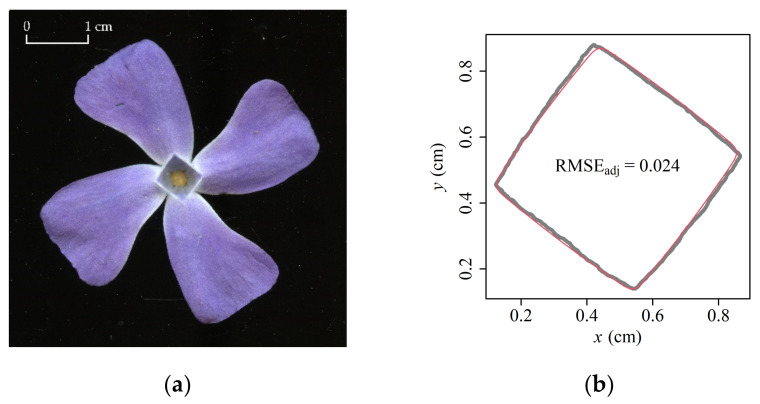
The vertical projection of *V. major* flower with four petals (**a**), and the comparison between the observed and predicted ORCTs (**b**). In panel (**b**), the gray curve represents the observed ORCT and the red curve represents the predicted ORCT using Model 2. Presentation and symbols as in Figure 3.

In addition to pentagonal ORCTs, there was a very small proportion of quadrangular ORCTs among the flowers of the sampled *V. major* population (Figure 6a). In addition, flowers with hexagonal ORCTs can be observed in this species (personal observations), but were not found in the current study. The plants with pentagonal ORCTs have five petals, while the plants with quadrilateral ORCTs have four petals; each angle of an ORCT corresponds to a petal (Figure 1and Figure 6). In flower sampling, we found only six specimens with quadrilateral petals, i.e., a ratio of 6:366 in the total sample of flowers collected. It is currently unclear what causes the formation of four-petal flowers in *V. major*, but given that it is a clonal species, it is unlikely that the variation of petal number is of genetic origin, although we cannot rule out that the sampled population was a mix of several clones. In fact, the situation is similar to *Syringa* spp. that typically have four-petal flowers, but occasionally form five-petal flowers, and sometimes even flowers with more than five petals in the same inflorescence.

Similar to flowers with pentagonal ORCTs, we fitted the coordinate data of the six quadrilateral ORCTs of *V. major* using Model 2. As with flowers with pentagonal ORCTs, all of the adjusted RMSE values for flowers with quadrilateral ORCTs were smaller than 0.05 (Table S3 in the online Supplementary Materials). Thus, Model 2 also well describes the shape of the ORCT of *V. major* with four petals (Figure 6b).

## 5. Conclusions

The simplified Gielis equations with *m* = 5 or *m* = 4 are both valid in describing the outer rims of the corolla tubes (in top view) (ORCTs) of *V. major*. A further simplified Gielis model with *n*_2_ = *n*_3_ predicted a perfectly axial symmetrical ORCT. The use of the simpler model can avoid model overfitting and reduce the running time for completing parameter estimation. This work shows that the ORCTs of plants can be modeled by the Gielis equation and can provide a reference for future research on superelliptic shapes of plant organs or tissues, e.g., projections of calyxes and polygonal cross-sections of fruits, e.g., ref. [9]). Additionally, the small difference between the parameters *n*_2_ and *n*_3_ in Model 1 did not cause a large deviation for ORCTs from a perfectly axial symmetrical pattern. This work provides evidence that the natural morphology of plant organs and tissues follows the superformula proposed by Gielis [2], and the process of growth and development of corolla tubes exhibits a general but unknown biophysical mechanism that is commonly shared by other organs or tissues of many species, which at least can be reflected by the same mathematical model. Thus, it is worth further examining the validity of the Gielis equation in depicting more geometries found in nature to uncover the influence(s) of such a general biophysical mechanism on biological geometry and morphology.

## Data Availability

The data can be found in Tables S1–S3 in the online Supplementary Materials.

## Supplementary Materials

The following supporting information can be downloaded at: https://www.mdpi.com/article/10.3390/plants11151987/s1; Table S1: Fitted results of the planar coordinate data of ORCTs of *V. major* with five petals using Model 1; Table S2: Fitted results of the planar coordinate data of ORCTs of *V. major* with five petals using Model 2; and Table S3: Fitted results of the planar coordinate data of ORCTs of *V. major* with four petals using Model 2.
